# Editorial: New trends in osteoarthritis treatment

**DOI:** 10.3389/fmed.2024.1372052

**Published:** 2024-01-31

**Authors:** Assunta Pozzuoli, Elisa Belluzzi, Pietro Ruggieri

**Affiliations:** ^1^Musculoskeletal Pathology and Oncology Laboratory, Department of Surgery, Oncology and Gastroenterology DiSCOG, University of Padova, Padova, Italy; ^2^Orthopedics and Orthopedic Oncology, Department of Surgery, Oncology and Gastroenterology DiSCOG, University of Padova, Padova, Italy; ^3^Centre for Mechanics of Biological Materials, University of Padova, Padova, Italy

**Keywords:** osteoarthritis, knee, treatment, extracellular vesicles, articular cartilage, regenerative rehabilitation, autophagy, mitochondria

Osteoarthritis (OA) is the most common type of arthritis affecting millions of persons worldwide (1, 2). It is a complex and multifactorial disease that could affect any joint, but particularly the knee, hip and hands. All the joint tissues are involved, including synovial membrane, subchondral bone, infrapatellar fat pad, subchondral bone, and especially cartilage, which undergoes several changes impacting its biomechanical behavior (3–5). These changes lead to swelling, pain, and difficulty in joint movement, thus impacting quality of life (6).

Several risk factors have been identified such as joint injury, comorbidities, female gender, genetic predisposition, obesity, metabolic diseases, and age (7, 8).

Despite the high prevalence of OA, there is still no treatment to cure or delay the progression of OA.

Currently, medical treatment focuses on symptoms relief with painkillers and anti-inflammatory drugs to improve patient's quality of life (9, 10). This Research Topic aimed to address new trends and updates in OA treatment, including pharmaceutical, non-pharmaceutical, and surgical treatments. A total of 11 articles were published: 2 reviews, 1 systematic review, 1 scoping review, 1 clinical trial, and 6 original research articles on OA treatments.

Dyslipidaemia represents a risk factor for OA onset and progression. It is usually treated with statins, drugs generally safe and well tolerated. While statins efficacy in reducing cardiovascular diseases is well documented, their effect on skeletal muscles is poor investigated. As statin-induced muscle symptoms have been reported as a cause of statin discontinuation, Lim et al. performed a *post-hoc* analysis of a placebo-controlled trial to evaluate the effect of atorvastatin on skeletal muscles of patients with knee OA. Only a tendency for increased myalgia was reported not clearly related to atorvastatin.

Recently, new lipid-lowering drugs are used in the secondary prevention of atherosclerosis but their effects on OA have not been reported. Wang et al. estimated the casual effects of blood lipids and lipid-lowering agents on knee and hip OA risk, performing a Mendelian randomization study. A genetic predisposition to higher blood LDL-C levels may decrease the risk of knee and hip OA, independently of HDL-C and TG levels, and body mass index (BMI). Moreover, genetically proxied LDL-C-lowering effects of statins increased the risk of knee but not hip OA.

Female gender and obesity are well-documented risk factors for OA. OA incidence increases in women after menopause due to oestrogens decrease, weight gain, and BMI increase. Based on this evidence, Abshirini et al. performed a placebo-controlled trial enrolling 55 overweight/obese postmenopausal women with joint discomfort at risk or at early-stage OA. They evaluated the effect of whole greenshell mussel (GSM) powder, supplemented for 12 weeks, on biomarkers of cartilage metabolism, inflammatory cytokines, and joint symptoms and functions. Oral GSM supplementation was effective in improving overall joint pain and it might slow down type II collagen degradation but did not impact on knee-related symptoms and on the level of inflammatory cytokines, suggesting that GSM may act within the joint microenvironment rather than at the systemic level.

Genetic predisposition plays a role as a risk factor for OA along with ethnic heritage and geographic localization (11). Several genome-wide association studies investigated the relationship between fat mass and obesity-related (FTO) gene variation and OA risk but with inconclusive results. Therefore, Zhao et al. conducted an integrated meta-analysis with bioinformatics to better elucidate the role of the FTO gene in the development of OA, confirming that FTO gene polymorphism increased OA risk especially through obesity in the Caucasian population.

Several treatments have been proposed especially in early-stages of OA, including cells and extracellular vesicles (EVs) therapies (12, 13). Colombini et al. applied a bioinformatics approach to study the miRNA composition of EVs secreted by cartilage cells (CCs), adipose tissue-derived (ASCs), and bone marrow-derived stem cells (BMSCs), isolated from hip OA patients. Moreover, the authors co-cultured CCs, ASCs, and BMSCs with T cells and macrophages showing immunomodulatory ability, supporting the rationale behind the use of cell-based therapy for OA treatment.

Regenerative rehabilitation, which involves both regenerative and rehabilitation medicine, is a new approach for OA treatment (14). Popov et al. applied a regenerative rehabilitation mathematical model of local articular cartilage defects based on the features of cartilage tissue and the responses of chondrocytes and progenitor chondrocytes observed in *in vitro* experiments applying different mechanical stimuli. Tissue micro and macro environment, restored after mechanical stimulation, had a significant effect on ECM formation of cartilage.

The use of a disease modifying treatment for OA is a growing area of interest. Lin et al. used a comprehensive 3D contrast enhanced μCT to evaluate the effect of intra-articular injection of a micronized dehydrated human amnion/chorion membrane (mdHACM) on joint tissues in a preclinical post-traumatic OA rat model. mdHACM was delivered intra-articularly 24 h (acute treatment) or 3 weeks (delayed treatment). Delayed treatment improved joint health, slowing the degeneration of cartilage, subchondral bone, and marginal osteophytes. This study supports the suitability of mdHACM to treat symptomatic OA.

Autophagy has a protective role against microenvironment changes in knee OA and its failure could worse cartilage degradation (15). Wu et al. showed the activation of autophagy in human chondrocytes and in cartilage of an OA rabbit model after intra-articular injection of clioquinol. Moreover, clioquinol had a protective effect increasing the expression of ECM components, suppressing inflammatory mediators and decreasing chondrocyte apoptosis.

Gene therapy is a growing Research Topic in OA treatment allowing local production of target therapeutic proteins (16). Uebelhoer et al. conducted a scoping review on the current knowledge about gene therapies in preclinical and clinical settings. Studies about *in vitro, in vivo*, or *ex vivo* gene therapies were analyzed. The results showed that gene therapy could be a highly promising treatment for OA.

Mitochondrial function could represent a target for OA treatment (17). Mao et al. reviewed mitochondrial dysfunctions in OA chondrocytes reported as decreased ATP production, increased oxidative stress, calcium dysregulation, increased permeability of the mitochondrial membrane, mtDNA alternations, and activation of mitochondrial apoptotic pathway resulting in cartilage degeneration. Endogenous mitochondrial molecular targets, exogenous drugs, stem cells and exosomes, that could improve mitochondrial function, were reviewed.

OA has been described as a “wound that does not heal” because of the dysregulation of the immune response, the inflammation, and the normal healing and repair process (18). Huston reviewed the positive effects of Tai Chi in OA treatment. Tai Chi can improve knee alignment, optimize knee biomechanical forces, strengthen the lower limbs, and importantly can decrease systemic inflammation. Moreover, Tai Chi is able to decrease the risk of falls and further injury of patients affected by OA.

Collectively, this Research Topic focused on the effects of new treatments for OA also discussing new possible targets. Different strategies were used, starting from clinical trials to *in-silico* models. Further studies are needed to find new treatments and test their efficacy and safety in controlled randomized clinical trials. In this context, research unraveling OA pathophysiological mechanisms is essential in order to better elucidate the complexity of this disease.

## Author contributions

AP: Writing – original draft, Writing – review & editing. EB: Writing – original draft, Writing – review & editing. PR: Writing – original draft, Writing – review & editing.
